# Association Between FOXP3 rs2232368 Variant and Hashimoto’s Thyroiditis Risk: A Case-Control Study

**DOI:** 10.7759/cureus.79126

**Published:** 2025-02-16

**Authors:** Sarah Kassab Shandaway Al-Zamali, Iman Mohammad Said Jallod, Shahad Saad Mohammed, Mohammed Sameir

**Affiliations:** 1 Medical Microbiology, Hammurabi College of Medicine, University of Babylon, Hillah, IRQ; 2 Basic Science Nursing, College of Nursing, University of Telafer, Telafer, IRQ; 3 Public Health Sciences, Technical Institute of Babylon, Al-Furat Al-Awsat Technical University, Najaf, IRQ; 4 Clinical Autoimmune Therapy, Hammurabi College of Medicine, University of Babylon, Hillah, IRQ

**Keywords:** autoimmune disorder, foxp3 gene, hashimoto's thyroiditis, regulatory t cell, rs2232368 polymorphism

## Abstract

Background and aim

Hashimoto’s thyroiditis (HT) pathogenesis is characterized by a dysregulation of immune tolerance, which may be influenced by genetic variations in the FOXP3 gene, a key regulator of T-regulatory cell function. However, the role of specific FOXP3 polymorphisms in HT susceptibility is not yet fully understood, particularly in Middle Eastern populations. This study aims to explore the association between the FOXP3 rs2232368 polymorphism and HT susceptibility in an Iraqi population while also examining its relationship with thyroid function parameters.

Methods

This case-control study included 60 HT patients and 40 healthy controls from the Medical City Educational Laboratories in Baghdad (October 2022 to September 2023). HT diagnosis was based on established clinical and laboratory criteria. Thyroid function (thyroid-stimulating hormone (TSH), triiodothyronine (T3), and thyroxine (T4)) was measured using the mini VIDAS^®^ system (bioMérieux, Craponne, France). FOXP3 rs2232368 genotyping was performed using ARMS-PCR. Genetic associations were assessed through ORs with 95% CIs, adjusting for demographic and clinical variables.

Results

HT patients exhibited significant thyroid dysfunction compared to controls (median TSH: 18.82 vs. 2.66 mIU/L, p < 0.001; T3: 0.53 vs. 2.33 nmol/L, p < 0.001; T4: 8.12 vs. 43.5 μg/dL, p < 0.001). The AA genotype was associated with a significantly increased risk of HT (OR = 4.66, 95% CI: 1.32-16.44, p = 0.017), while the heterozygous GA genotype showed a nonsignificant trend (OR = 2.28, 95% CI: 0.84-6.19, p = 0.108). The A allele was strongly associated with HT susceptibility (OR = 2.98, 95% CI: 1.54-5.77, p = 0.001). These associations remained significant after adjusting for BMI and thyroid parameters.

Conclusions

This study identifies FOXP3 rs2232368 as a significant genetic risk factor for HT in the Iraqi population, with the AA genotype associated with nearly a five-fold increased susceptibility. These findings enhance our understanding of the genetic basis of HT and may inform risk stratification strategies for Middle Eastern populations. Further research is needed to explore the functional implications of this polymorphism in thyroid autoimmunity.

## Introduction

Hashimoto’s thyroiditis (HT) is the most common organ-specific autoimmune disorder, with a global prevalence of 0.3-2% and a strong female preponderance (female-to-male ratio: 7-10:1). In this condition, the immune system attacks the thyroid gland, causing inflammation and leading to hypothyroidism, which is the leading cause of hypothyroidism in iodine-sufficient areas [1]. The natural history of HT follows a complex course, with distinct phases: initial thyrotoxicosis due to follicular disruption, transient euthyroidism maintained by compensatory mechanisms, and eventual hypothyroidism resulting from extensive tissue destruction [2]. Diagnosis involves measuring blood levels of thyroid hormones (triiodothyronine (T3) and thyroxine (T4)) and thyroid-stimulating hormone (TSH), as well as thyroid antibody tests. The risk of developing papillary thyroid cancer is 50 times greater in HT patients, while the risk of primary thyroid lymphoma is reportedly 60 times higher than in the general population [3].

The pathogenesis of HT results from a complex interplay between genetic susceptibility and environmental factors, driven by multiple immunological mechanisms [4,5]. A key factor in this process is the dysregulation of immune tolerance, particularly involving regulatory T cells (Tregs), which normally maintain immune homeostasis and prevent autoimmune responses [6,7].

Ultimately, the combination of these factors, along with chronic inflammation and progressive thyroid gland destruction, leads to hypothyroidism [8]. Tregs, which help maintain tolerance and protect against autoimmune diseases [9], are thought to play a crucial role. Individuals with HT may have defective or insufficient Tregs, leading to the expansion of self-reactive immune cells and the destruction of the thyroid [10].

The FOXP3 gene, located at Xp11.23, encodes a 431-amino-acid transcriptional repressor that is essential for Treg development and function. Through intricate molecular mechanisms, FOXP3 regulates the suppressive functions of Tregs by modulating cytokine expression and cellular activation pathways [9]. Genetic variations in FOXP3, particularly single nucleotide polymorphisms (SNPs), may influence autoimmune susceptibility by altering Treg function [10]. The rs2232368 polymorphism has been identified as a potentially significant variant, with associations observed in various autoimmune conditions, including asthma and breast cancer [11,12].

Despite these findings, the specific role of the FOXP3 rs2232368 polymorphism in HT susceptibility remains poorly understood, especially across different ethnic populations. This gap in knowledge is particularly evident in Middle Eastern populations, where unique genetic and environmental factors may influence disease susceptibility. Therefore, this study aims to explore the association between the FOXP3 rs2232368 polymorphism and HT susceptibility in an Iraqi cohort, with the goal of identifying population-specific genetic risk factors and potentially discovering novel therapeutic targets. This investigation will contribute to a deeper understanding of HT pathogenesis and may guide personalized therapeutic strategies based on genetic profiling.

## Materials and methods

Study design and population

This case-control study assessed the association between FOXP3 gene polymorphisms and HT susceptibility in an Iraqi cohort. The research was conducted at the Educational Laboratories, Medical City, Baghdad Governorate, Iraq, from October 2022 to September 2023. The study protocol was approved by the Public Health Directorate, Ministry of Health, Republic of Iraq (approval number 411, dated 2021/04/25). Written informed consent was obtained from all participants in accordance with the principles of the Helsinki Declaration.

Sample size and subject selection

Sixty newly diagnosed HT patients and 40 healthy controls were enrolled, with frequency matching for age (±5 years) and sex (female-to-male ratio: 7:1). The sample size was determined using G*Power software (version 3.1.9.4), assuming an effect size of 0.5, α = 0.05, and power = 0.8. HT diagnosis was confirmed through clinical examination and laboratory tests by board-certified endocrinologists, following standard diagnostic criteria (appropriate guidelines cited).

Inclusion and exclusion criteria

The inclusion criteria for HT patients consisted of a confirmed diagnosis of HT based on clinical presentation and laboratory parameters, an age range of 18-65 years, and no prior use of thyroid medication. Control subjects were selected to match the age and sex demographics, with normal thyroid function and no personal or familial history of autoimmune diseases. Exclusion criteria for both groups included concurrent autoimmune conditions, inflammatory diseases, malignancies, pregnancy, and the use of immunomodulatory medications. All participants underwent a standardized clinical assessment, and their medical histories were thoroughly documented.

Thyroid function assessment

Thyroid hormones (TSH, T3, and T4) were measured using the Mini VIDAS® immunoanalyzer (bioMérieux, Craponne, France), following the manufacturer’s protocols. The assay specifications included analytical sensitivities of TSH: 0.05 mIU/L, T3: 0.2 nmol/L, and T4: 0.5 μg/dL, with intra-assay CV <5% and inter-assay CV <7%. Quality control samples (low, medium, and high) were included in each analytical run, and reference ranges were determined according to laboratory standards.

FOXP3 rs2232368 genotyping

Genotyping was performed using the Amplification Refractory Mutation System PCR (ARMS-PCR). Primers were designed with the Primer1 ARMS PCR tool, based on sequences from the NCBI SNP database (Table 1). PCR reactions (25 μL) consisted of 12.5 μL G2 Green Master Mix, 2 μL of each primer (10 pmol), 5 μL template DNA, and 3.5 μL nuclease-free water. Amplification was carried out in a Bio-Rad thermal cycler (Bio-Rad, Hercules, California, USA) under optimized conditions: initial denaturation at 95°C for five minutes, followed by 35 cycles of denaturation at 95°C for 30 seconds, annealing at 60°C for 30 seconds, extension at 72°C for 30 seconds, and a final extension at 72°C for five minutes.

**Table 1:** ARMS-PCR primers ARMS-PCR, Amplification Refractory Mutation System PCR

Primers	Sequence base (5′-3′)	Amplicon size
FOXP3 rs2232368 G allele reverse wild-type primer	AGTGCCTAAGTAGGGAGAAG ATTAC	149 bp
FOXP3 rs2232368 A allele reverse mutant-type primer	AGTGCCTAAGTAGGGAGAAG ATTAT	
FOXP3 common forward primer	TGAGAGGGAGACTGAGGTA G	

DNA extraction and quality assessment

Genomic DNA was extracted from EDTA-anticoagulated blood using the gSYAN extraction kit (Geneaid Biotech Ltd., New Taipei City, Taiwan), following the manufacturer’s instructions. DNA quantity and quality were assessed with a Thermo Nanodrop spectrophotometer (Thermo Fisher Scientific Inc., Waltham, Massachusetts, USA). Samples with A260/280 ratios between 1.8 and 2.0 and concentrations greater than 50 ng/μL were selected for genotyping.

ARMS-PCR genotyping protocol

Genotyping of the FOXP3 (rs2232368) G/A polymorphism was performed using the ARMS-PCR technique. Two separate sets of tubes were prepared based on the number of samples: one set for the wild-type allele and another for the mutant allele. The wild-type forward primer was added to the first set of tubes, while the mutant forward primer was added to the second set. Each PCR reaction was performed in a total volume of 25 μL, consisting of 12.5 μL of G2 Green Master Mix, 2 μL of forward primer (10 pmol, specific to either the wild-type or mutant allele), 2 μL of a common reverse primer (10 pmol), 5 μL of DNA template, and 3.5 μL of PCR-grade water. The samples were then amplified in a Bio-Rad thermal cycler under the following conditions: initial denaturation at 95°C for five minutes, followed by 35 cycles of denaturation at 95°C for 30 seconds, annealing at 60°C for 30 seconds, and a final extension at 72°C for five minutes. After PCR, 5 μL of the ARMS-PCR products were mixed with an equal volume of loading dye and loaded onto a 2% agarose gel prepared with TBE buffer. The gel was stained with 0.5 μL of ethidium bromide, and electrophoresis was performed at 70V for approximately 50 minutes. The resulting DNA bands were visualized under UV light at 312 nm using a transilluminator, and gel images were captured. Amplicon sizes were compared to a DNA ladder for analysis. Amplification in only one tube indicated either a homozygous wild-type or homozygous mutant genotype, while amplification in both tubes indicated a heterozygous genotype.

The PCR protocol included an initial denaturation at 95°C for five minutes, followed by 35 cycles of denaturation at 95°C for 30 seconds, annealing at 60°C for 30 seconds, and a final extension at 72°C for five minutes. After PCR, 5 μL of the ARMS-PCR products were mixed with an equal volume of loading dye and loaded onto a 2% agarose gel prepared with TBE buffer. The gel was stained with 0.5 μL of ethidium bromide, and electrophoresis was run at 70V for approximately 50 minutes. DNA bands were visualized under UV light at 312 nm using a transilluminator, and gel images were captured. Amplicon sizes were compared to a DNA ladder for analysis. Amplification in only one tube indicated either a homozygous wild-type or homozygous mutant genotype, while amplification in both tubes indicated a heterozygous genotype. Gel images were analyzed by comparing the amplicon sizes to the ladder, and amplification in one tube confirmed either the homozygous wild-type or mutant genotype, whereas amplification in both tubes indicated a heterozygous genotype.

Statistical analysis

Data analysis was conducted using IBM SPSS Statistics for Windows, Version 25.0 (Released 2017; IBM Corp., Armonk, NY, USA). Normality was assessed with the Shapiro-Wilk test. Continuous variables were expressed as median (IQR) or mean ± SD, as appropriate. Categorical variables were compared using the χ² test. Genotype and allele frequencies were analyzed using Fisher’s exact test. The strength of associations was estimated using ORs with 95% CIs. Hardy-Weinberg equilibrium was assessed in the control group. Statistical significance was set at p < 0.05 (two-tailed).

## Results

Characteristics of the study population

This case-control study included 100 participants: 60 patients with HT and 40 healthy controls (Table 1). As shown in Table 2, the demographic analysis revealed no significant differences between the groups in terms of age (HT: 37.17 ± 8.02 years; controls: 38.85 ± 7.87 years; p = 0.453) and gender distribution (HT: 90.0% female; controls: 75.0% female; p = 0.077). However, BMI analysis showed significantly higher values in HT patients compared to controls (29.57 ± 6.52 vs. 24.11 ± 3.80 kg/m²; p < 0.001).

**Table 2:** Demographic characteristics, BMI, and thyroid function test results of the studied groups T3, triiodothyronine; T4, thyroxine; TSH, thyroid-stimulating hormone

Characteristic	Patients (n = 60)	Healthy controls (n = 40)	p-Value
Age (years)	37.17 ± 8.02	38.85 ± 7.87	0.453
Sex (male/female)	6 (10.0%)/54 (90.0%)	10 (25.0%)/30 (75.0%)	0.077
BMI (kg/m²)	29.57 ± 6.52	24.11 ± 3.80	<0.001
TSH (mIU/L)	18.82 ± 2.43	2.57 ± 0.78	<0.001
T3 (nmol/L)	0.53 ± 0.089	2.18 ± 0.067	<0.001
T4 (nmol/L)	8.12 ± 3.44	43.50 ± 2.39	<0.001

Thyroid hormone measurements

**Figure 1:**
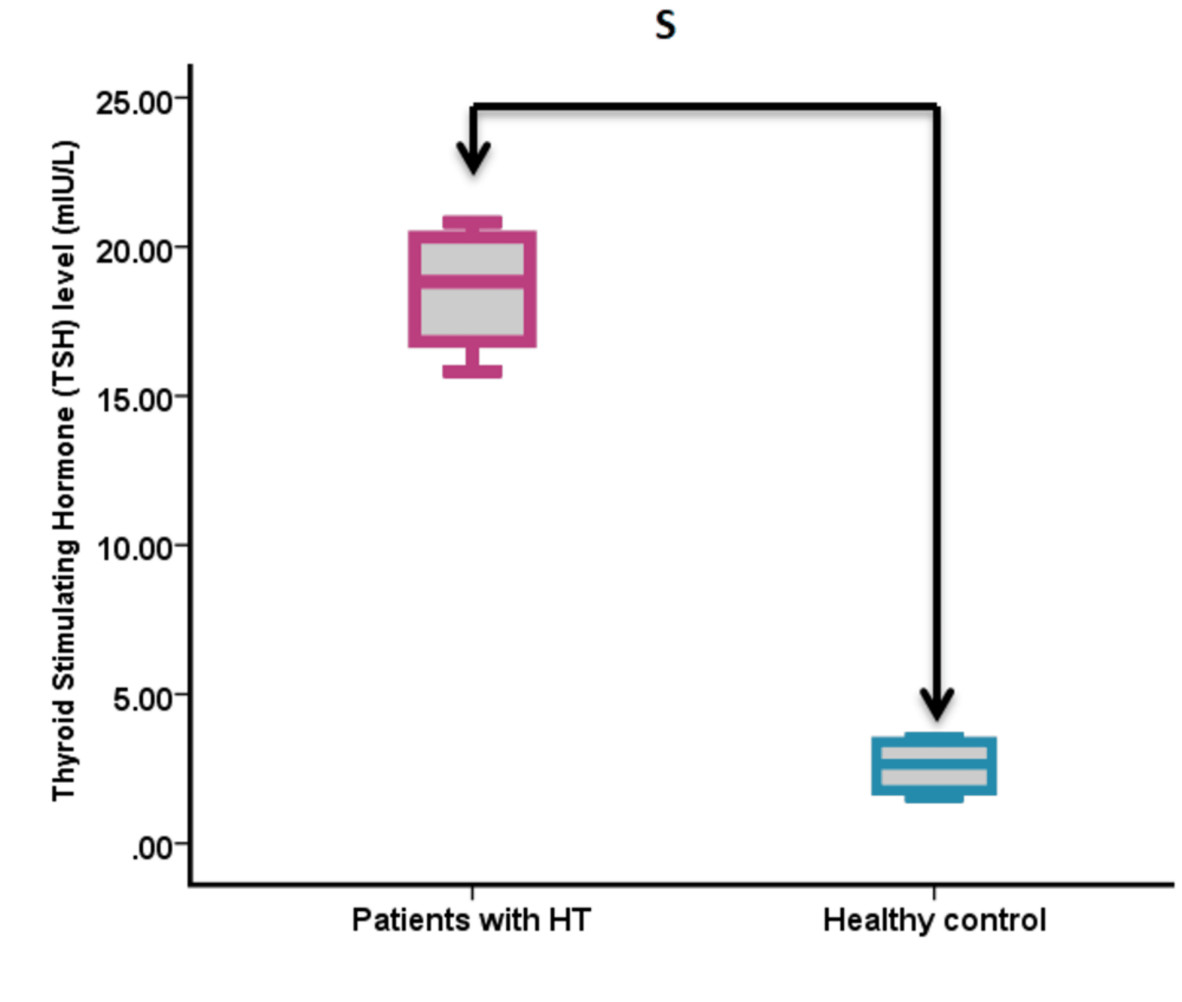
Serum TSH levels in patients with HT compared to healthy controls S = significance at p < 0.05 HT, Hashimoto’s thyroiditis; TSH, thyroid-stimulating hormone

**Figure 2:**
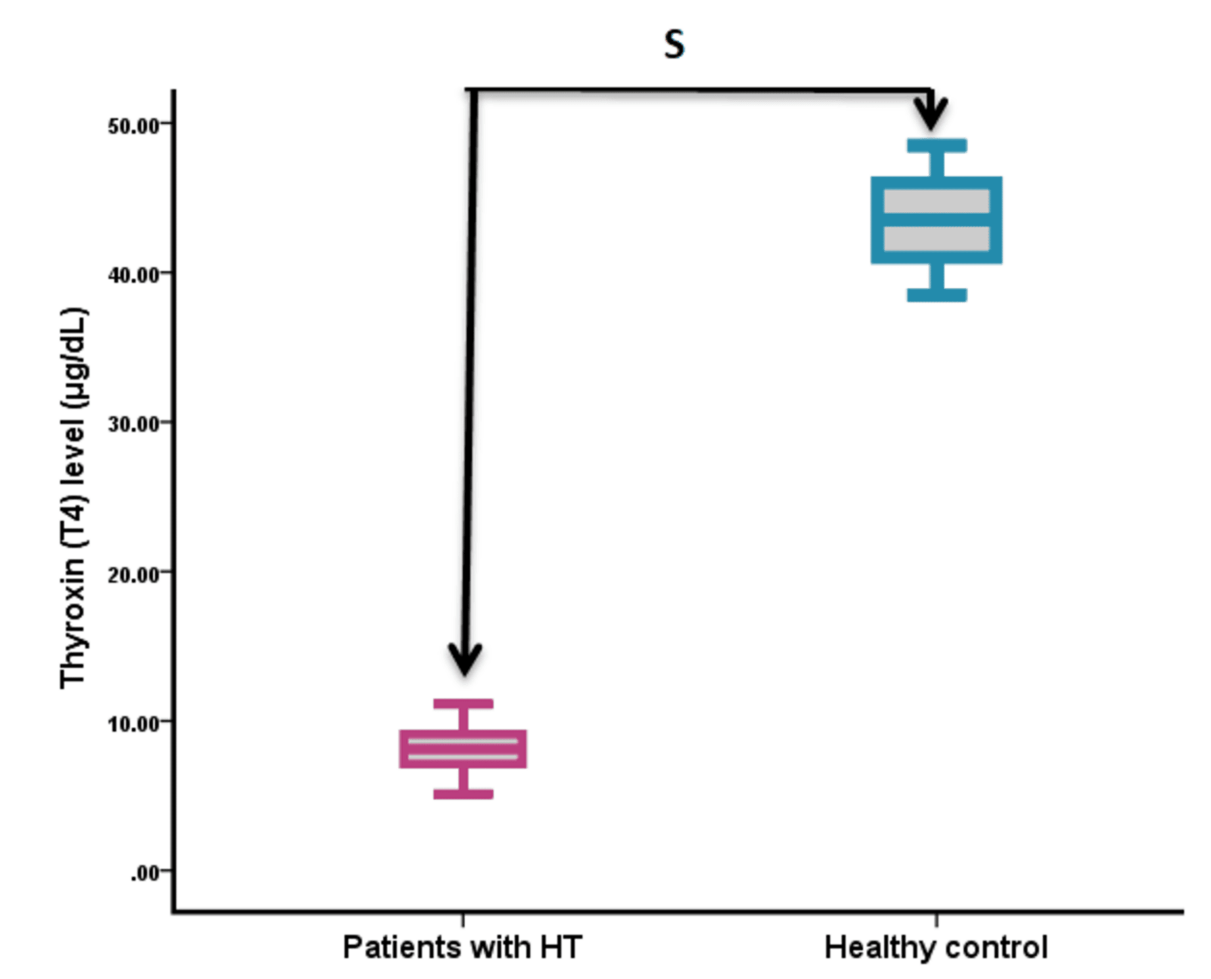
Serum T4 levels in patients with HT compared to healthy controls S = significance at p < 0.05 HT, Hashimoto’s thyroiditis; T4, thyroxine

**Figure 3:**
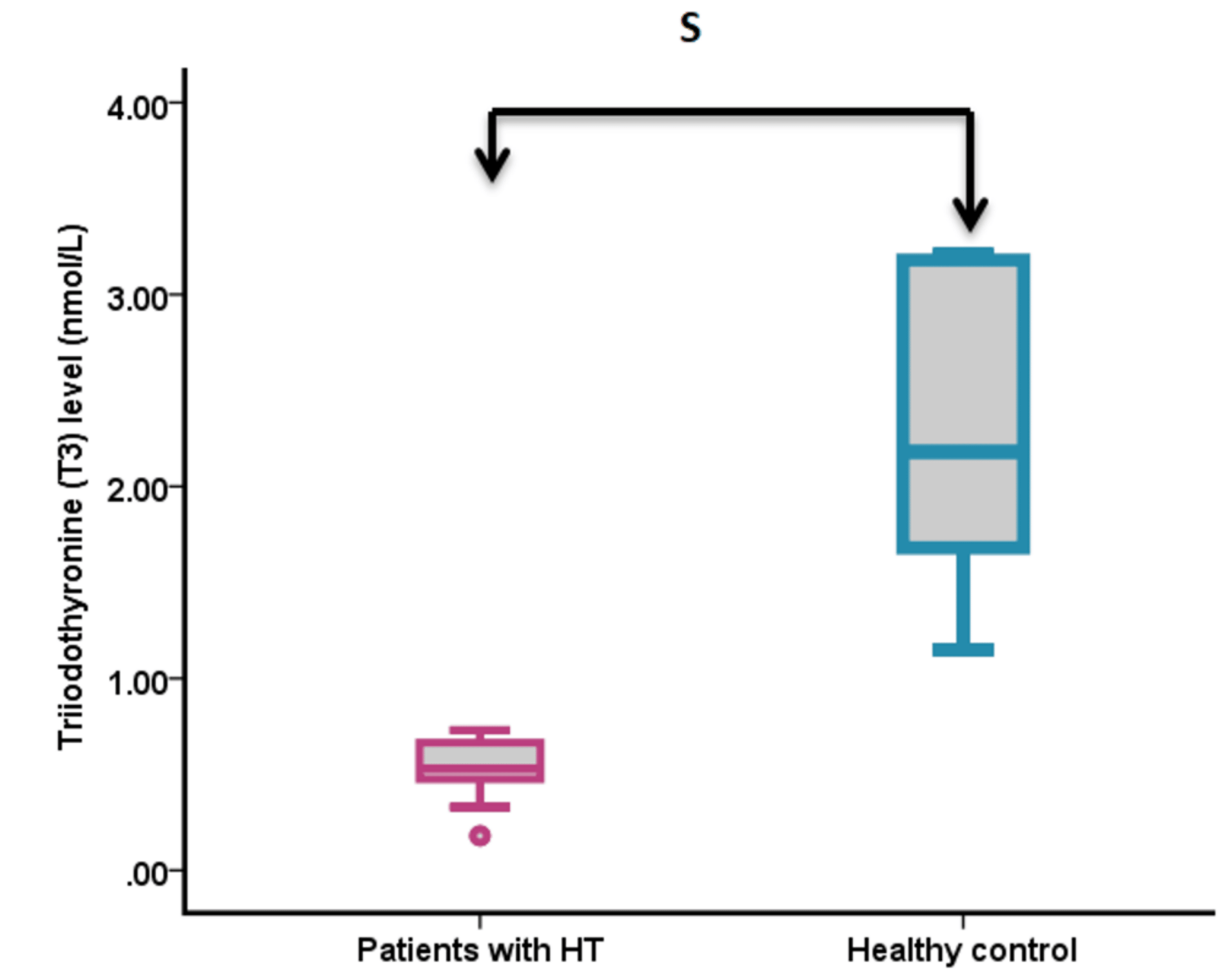
Serum T3 levels in patients with HT compared to healthy controls S = significance at p < 0.05 HT, Hashimoto’s thyroiditis; T3, triiodothyronine

Analysis of thyroid function parameters revealed significant differences between the groups (Figure 1, Figure 2, Figure 3). TSH levels were significantly higher in HT patients compared to controls (18.82 ± 2.43 vs. 2.57 ± 0.78 mIU/L; p < 0.001). In contrast, both T3 and T4 levels were significantly lower in HT patients than in controls (T3: 0.53 ± 0.089 vs. 2.18 ± 0.067 nmol/L, p < 0.001; T4: 8.12 ± 3.44 vs. 43.50 ± 2.39 nmol/L, p < 0.001).

Molecular analysis of FOXP3 rs2232368 polymorphism

**Figure 4:**
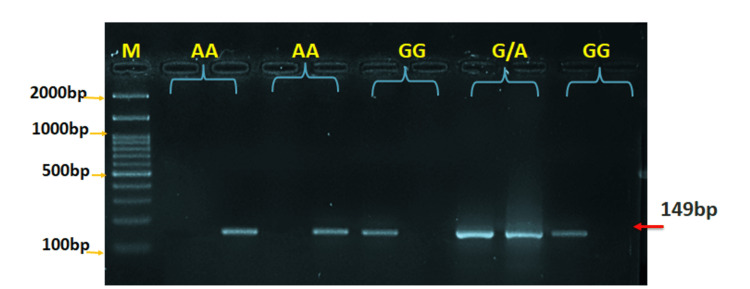
Agarose gel electrophoresis of ARMS-PCR products for FOXP3 rs2232368 (G/A) gene polymorphism in patients Wild-type homozygotes (GG) show amplification of the G allele only, while mutant homozygotes (AA) display amplification of the A allele only. Heterozygotes (GA) exhibit both G and A alleles. Marker (M) range: 2,000-100 bp ARMS-PCR, Amplification Refractory Mutation System PCR

Genotyping using ARMS-PCR revealed three distinct genotypes at the FOXP3 rs2232368 locus: wild-type homozygous (GG), heterozygous (GA), and variant homozygous (AA). Electrophoretic separation showed distinct banding patterns for each genotype: the GG genotype exhibited amplification of the wild-type G allele, the AA genotype showed amplification of the variant A allele, and the GA genotype displayed both allelic patterns (Figure 4). Genotype distribution analysis confirmed Hardy-Weinberg equilibrium in both the patient and control groups, supporting the genetic stability of the study population.

Genotypic and allelic distribution analysis

A comprehensive analysis of the FOXP3 rs2232368 polymorphism revealed significant associations with HT susceptibility (Table 3). Under the co-dominant model, the homozygous variant genotype (AA) was associated with a significantly higher risk of HT development (OR = 4.66, 95% CI: 1.21-17.92, p = 0.017) compared to the wild-type homozygous genotype (GG). The heterozygous genotype (GA) showed an elevated but nonsignificant association with HT risk (OR = 2.28, 95% CI: 0.82-6.35, p = 0.108). Genetic model analysis revealed significant associations under both dominant and recessive models. The dominant model (AA+GA vs. GG) showed a significant disease association (p = 0.012), while the recessive model (AA vs. GA+GG) confirmed increased HT susceptibility (OR = 3.75, 95% CI: 1.03-14.04, p = 0.038). At the allelic level, the variant A allele was strongly associated with HT risk (OR = 2.98, 95% CI: 1.48-6.01, p = 0.001) compared to the wild-type G allele, with frequencies of 36.7% in patients versus 16.3% in controls.

**Table 3:** FOXP3 (rs2232368) POLY genotype frequency in patients and healthy controls ¥ = Chi-square test; S = significant at p < 0.05

Mode	FOXP3 (rs2232368)	Patients (n = 60)	Healthy controls (n = 40)	p-Value	OR	95% CI
Co-dominant	AA	14 (23.3%)	3 (7.5%)	0.017	4.66	1.21-17.92
	G/A	16 (26.7%)	7 (17.5%)	0.108	2.28	0.8-6.35
	GG	30 (50.0%)	30 (75.0%)	Reference		
Dominant	AA + G/A	30 (50.0%)	10 (25.0%)	0.012^¥,S^	Reference	
	GG	30 (50.0%)	30 (75.0%)		0.333	0.113-0.80
Recessive	AA	14 (23.3%)	3 (7.5%)	0.038^¥,S^	3.75	1.03-14.04
	G/A + GG	46 (76.7%)	37 (92.5%)		Reference	
Alleles	A	44 (36.7%)	13 (16.3%)	0.001^¥,S^	2.98	1.48-6.01
	G	76 (63.3%)	67 (83.7%)		Reference	

## Discussion

Demographic and clinical characteristics

HT is an autoimmune disorder primarily characterized by hypothyroidism, with heritability estimates ranging from 70-80% [13]. In our cohort, the average age of HT patients was 37.17 years, which is consistent with findings from Iraq [14] but contrasts with studies that report peak incidence in individuals aged 45-65 years [15,16]. These discrepancies could be attributed to differences in sampling methodologies, such as selection bias, regional diagnostic practices, or cohort demographics. As noted in previous literature [17-19], HT predominantly affects females, a trend commonly observed in autoimmune diseases. This sex disparity may be driven by X-linked immune regulatory genes or gene-environment interactions, such as hormonal influences or epigenetic modifications [20].

Metabolic and hormonal profiles

Our study revealed a significant difference in BMI between HT patients and healthy controls, suggesting a potential link between HT and overweight/obesity. Notably, there were no significant differences in age and sex distributions between the groups, indicating that the elevated BMI and hormonal imbalances (e.g., increased TSH and decreased T3/T4) observed in HT patients are independent of these variables. These metabolic and hormonal disruptions align with the hypothyroid state seen in HT and are consistent with prior findings [17,21-23]. Clinically, this underscores the importance of addressing BMI and thyroid hormone replacement as part of HT management.

Genetic associations with FOXP3 rs2232368

The rs2232368 SNP in the FOXP3 gene, which regulates the function of Tregs, was found to be associated with a five-fold increased risk of HT in individuals homozygous for the AA genotype. Further allelic analysis revealed a three-fold higher risk in carriers of the A allele. Although rs2232368 has been linked to various autoimmune and reproductive disorders - including recurrent spontaneous abortion in Han Chinese [24], breast cancer in Asian Indians [25], and asthma in Brazilians [26] - its role in HT remains underexplored. A 2024 meta-analysis confirmed its association with recurrent spontaneous abortion [27], but conflicting results across ethnic groups emphasize the need for population-specific studies. Given the critical role of FOXP3 in immune tolerance, variants like rs2232368 may disrupt Treg-mediated suppression of self-reactive lymphocytes, thereby increasing susceptibility to HT [28,29].

Strengths and limitations

This case-control study provides novel evidence linking rs2232368 to HT under a recessive model, offering potential insights for diagnostic and therapeutic strategies. However, the study design limits the ability to assess the role of this SNP in the progression of HT. Future research should aim to validate these findings in larger, multiethnic cohorts and explore the mechanistic links between FOXP3 variants, Treg dysfunction, and thyroid autoimmunity.

## Conclusions

This study highlights a significant association between the rs2232368 SNP in the FOXP3 gene and HT under a recessive model of inheritance. Individuals with the homozygous mutant AA genotype had a five-fold increased risk of developing HT compared to those with the wild-type GG genotype. Likewise, carriers of the A-allele exhibited a three-fold elevated risk relative to G-allele carriers. These results emphasize the potential role of FOXP3 genetic variants in HT susceptibility, likely through the dysregulation of Treg function and immune tolerance. However, larger, multiethnic studies are needed to confirm these findings and investigate the influence of ethnic diversity on the role of rs2232368 in HT. Such research is essential for improving diagnostic tools and developing therapeutic strategies based on the genetic and immunological mechanisms underlying autoimmune thyroid diseases.
